# Low back pain treatment adherence barriers in Eswatini private physiotherapy practices: A pilot study

**DOI:** 10.4102/sajp.v80i1.2077

**Published:** 2024-12-20

**Authors:** Tapiwa Chikaka, Monique M. Keller

**Affiliations:** 1 Department of Physiotherapy, Faculty of Health Sciences, University of the Witwatersrand, Johannesburg, South Africa

**Keywords:** adherence, barriers, low back pain, home exercise programme, physiotherapy, treatment

## Abstract

**Background:**

Adherence to management regimes is pivotal to successfully managing patients with low back pain (LBP). Barriers decrease adherence, resulting in disability.

**Objectives:**

Our pilot study aimed to determine barriers associated with physiotherapy treatment attendance and home exercise programme adherence among patients with LBP and treating physiotherapists in Eswatini.

**Method:**

A cross-sectional descriptive pilot study using a self-developed Research Electronic Data Capture (REDCap) survey was conducted with 62 LBP patients. Descriptive, regression and bivariate statistical analyses with odds ratios (OR) were used to identify adherence barriers.

**Results:**

Twenty-two (35.5%) participants missed appointments because of feeling better. Twenty-one participants (33.9%) missed scheduled appointments due to painful sessions, cost and the burden of changing their routines. Home exercise adherence was positively associated with understanding the condition (*p* = 0.001) and negatively with too many exercises (*p* = 0.005).

**Conclusion:**

Our study identified patient barriers to physiotherapy adherence, particularly females aged 40–69 years. Although doctor referrals improved adherence, time constraints and pain remained significant barriers. Barriers to prescribed home exercise programme adherence included pain during exercises, fear, no time, forgetting/no reference and too many exercises. While most participants understood their condition, some lacked understanding, underscoring the need for better education. Addressing these barriers could enhance adherence and reduce the impact of LBP.

**Clinical implications:**

To overcome the barrier of adherence to keeping appointments, prioritising health education and providing reasons for exercises should be empathised. Exercise programme adherence could be improved by providing reference material, less and more meaningful and/or functional exercises.

## Introduction

Low back pain (LBP) is a prevalent complaint, with higher rates in Africa, ranging between 39% and 47%, compared to global estimates (Morris et al. 2018). It impacts work performance and quality of life (Grabovac & Dorner 2019; Odole & Olugbenga-Alfred 2018). This results in increased medical consultations and the potential for treatment non-adherence (Hartvigsen et al. 2018). Middle- and low-income countries are primarily affected, with a 54% global increase in reported LBP from 1990 to 2015 (Hartvigsen et al. 2018). This is mainly driven by age and population growth (Hartvigsen et al. 2018). Low back pain frequently results in disability (Doualla et al. 2019). Disability reflects the negative interaction between health, personal and environmental factors (O’Young, Fosney & Ahn 2019).

Low back pain is a common reason for physiotherapy visits (Hartvigsen et al. 2018), with various effective treatment modalities (Lonsdale et al. 2012) such as hydrotherapy, electrotherapy, manual therapy and exercise therapy tailored to individual presentations (Gardner et al. 2017). The physiotherapist determines the treatment course and modalities used (Maher, Underwood & Buchbinder 2017) after a rigorous assessment of the condition and nature of the injury to eliminate pain, restore function and prevent future recurrences. Adherence to physiotherapy visits and treatment programmes is challenging.

According to the World Health Organization (WHO), adherence is described as ‘the extent to which a person’s behaviour taking medication, following a diet, or executing a lifestyle change corresponds with agreed recommendations from health care providers’ (WHO 2003). Failure to adhere to the guidelines of the physiotherapy treatment as recommended by the physiotherapist in charge has resulted in problems such as recurrent pain, work absenteeism, disability and high hospital costs (Babatunde, MacDermid & MacIntyre 2017).

Adherence to physiotherapy treatment for LBP faces various barriers (Al-Eisa 2010). Patients in semi-structured interviews in a Belgium study on chronic LBP commonly reported factors such as fatigue, a lack of motivation, pain-related fears and practical issues such as costs, equipment and support as reasons for non-compliance (Mathy et al. 2015). Physiotherapist-related barriers to patient adherence included speech, conduct, treatment choices and applications (Ali & May 2017). Communication skills impact the patient-to-physiotherapist relationship and can determine whether the patient feels comfortable continuing with physiotherapy and attending the follow-up treatments (Lonsdale et al. 2012). Delayed consultations have been identified as a significant factor contributing to poor outcomes in the management of LBP (Chen et al. 2018). This underscores the importance of timely medical consultations and adherence to recommended treatment schedules to improve patient outcomes (Chen et al. 2018). Providing information to the patients for them to understand their diagnosis, the purpose of the treatment procedure, the treatment programme and follow-up sessions, enhance patients’ adherence (Babatunde et al. 2017). Patients do remember the advice from their physiotherapists when shared decision-making was used during the first consultation, prescribed exercises were simple to do and few, patients’ concerns about their diagnosis were addressed and patients’ expectations were identified and addressed (Bachmann, Oesch & Bachmann 2018; Supp et al. 2020). There is an association between physiotherapists’ work setting and their clinical experience with LBP, which influences their attitudes towards managing this condition. Specifically, physiotherapists working in different settings (e.g. hospitals, private practices, sports clinics) and with varying levels of experience may develop distinct perspectives and approaches to treating LBP. This can affect their treatment decisions, patient interactions and overall management strategies (Roitenberg 2019). In managing LBP, physiotherapists are influenced by professional and organisational-level factors such as workload and knowledge (Perreault et al. 2018).

Health literacy is also related to adherence because it can significantly enhance treatment adherence by empowering patients with the knowledge and skills needed to manage their condition effectively. Health literacy is an individual’s ability to make the right health decision by understanding the basic health information and health services available to address the health condition; moreover, low literacy is associated with adverse health outcomes (Ratzan & Parker 2006).

Weather, time constraints, and increasing pain can hinder home-based exercise programmes, as was seen in a cross-sectional observational study of 51 participants (Van Koppen et al. 2016). In a French qualitative study with 29 patients presenting with chronic LBP, prescribed exercises, treatment plans, patient presentation and environmental factors affected adherence (Palazzo et al. 2016). A systematic review reported that minimal physical activity at baseline resulted in poor adherence; with low self-efficacy, depression, anxiety, social support, perceived barriers to exercise and pain levels during exercise as contributing factors (Jack et al. 2010). In India, geriatric patients encounter several obstacles, including location, clinic accessibility, travel difficulties, expenses, a lack of confidence to return to exercises, inability to recall exercises, anxiety and depression, difficulty with activities of daily living and a lack of family support (Anjum & Sonia 2019). In contrast to Anjum and Sonia (2019), a Mexican study conducted by Saner et al. (2018) discovered that depression, anxiety and fear of activities did not predict non-adherence to a stability exercise programme for chronic LBP.

Establishing the factors contributing to non-adherence to physiotherapy appointments and prescribed home exercises assist in highlighting the challenges patients face, especially with the paucity of studies in Eswatini. Our study aimed to explore the barriers to physiotherapy treatment adherence related to patients, physiotherapists and physiotherapy treatment institutions.

## Research methods and design

### Study design and participants

A cross-sectional descriptive pilot survey conducted in the Kingdom of Eswatini included patients attending private outpatient physiotherapy clinics. Inclusion criteria were males and females aged 18 years and above presenting with LBP, attending their second or more visits and treating physiotherapists. The exclusion criteria included pregnancy, neurological impairments, formally diagnosed psychiatric conditions and LBP with pain in another joint, for example, hip pain. The participants were identified from private physiotherapy clinics in Hhohho and Manzini. Six private physiotherapy outpatient facilities, out of a possible 11 identified clinics that provided informed consent, were included in our study.

### Instruments

The researcher developed a self-developed Research Electronic Data Capture (REDCap) survey, utilising published studies as a guide for data collection. Participant demographic information was obtained in the survey. The following survey sections comprised of two sections. One addressed the potential barriers to adherence related to patients, and the other the provider/institutional barriers to adherence, answered by the treating physiotherapist asking about provider-centred questions. The survey was translated into SiSwati and back-translated to English by an independent Eswatini national citizen to ensure consistency and accuracy in translation. The translation was performed by independent translators proficient in reading and writing in English and SiSwati. The survey content was developed from previous studies conducted on barriers to treatment adherence (Anjum & Sonia 2019; Babatunde et al. 2017; Bachmann et al. 2018; Chen et al. 2018; Jack et al. 2010; Lonsdale et al. 2012; Mathy et al. 2015; Palazzo et al. 2016; Perreault et al. 2018; Ratzan & Parker 2006; Roitenberg 2019; Saner et al. 2018; Supp et al. 2020; Van Dillen et al. 2016; Van Koppen et al. 2016). The validity and reliability of the survey were assessed by an expert (biostatistician) in the field. Readability, face validity, clarity and comprehensiveness were established.

### Sample size

A purposive sampling strategy was used to select our study population of patients and physiotherapists. In all, 100 patients agreed to participate.

### Study procedure

One clinic was selected using the drawing-out-of-the-hat method, after which the researcher requested patient records from the attending physiotherapists to identify potential participants. Diagnosis and patient notes were reviewed according to the inclusion criteria. From a 10% sample selection, our pilot testing included seven patients and one physiotherapist.

Patients’ contact details were used to contact the prospective participants. Patients were called for permission to use their personal information and participate in our study. Informed written consent was obtained before both the pilot testing and the main pilot study. Phone numbers were saved on the researcher’s smartphone. Survey links were sent via WhatsApp to those who had the WhatsApp application and via SMS (short message service) or email to those without the WhatsApp application. For participants present on the day, a hard copy survey was given, printed directly from REDCap survey or were offered a survey link via WhatsApp, where they completed the survey using their smartphones. The physiotherapists rendering care to the patients were asked to participate.

No changes were made to the survey after our pilot testing, and the survey was used to collect data for our main pilot study. The pilot testing participants were included in our main pilot study, and the data collection methods were the same in both our pilot testing and main pilot study.

Participants received reminders via the same platform that they received the first link. Reminders were sent within 2 weeks to 3 weeks if they did complete the survey. Data collection took 7 months, from April 2021 to November 2021.

### Data analysis

Data were exported from REDCap onto a Microsoft Excel spreadsheet. The data set was cleaned before data analysis. This process entailed checking whether the data set had missing values or irregular entries, and when missing values were identified participants were excluded from our study. Our study utilised the IBM Statistical Package for the Social Sciences (SPSS) version 20 statistical software (IBM Corporation, Armonk, New York, United States) for data analysis, employing descriptive statistics, regression analysis and bivariate analysis with odds ratios (OR). Statistical significance was set at *p* < 0.05.

### Ethical considerations

The researcher adhered to research ethical principles. Participants were protected by minimising the risk of harm, obtaining written informed consent, ensuring voluntary participation and maintaining confidentiality by using participant numbers instead of names. They were also given the right to withdraw from our study at any time without penalty. Misleading practices were avoided by providing clear and accurate information about our study’s purpose, procedures and potential risks, ensuring that participants fully understood what their involvement entailed. Data collection only commenced after obtaining ethical approval from the Human Research Ethics Committee at the University of Witwatersrand (Medical) with the number M201018, and from the Eswatini Health and Human Research Review Board with the number SHR 328-9/2021. Permission to conduct our study at the private clinics was obtained from the clinical directors or the department heads of the physiotherapy unit. Soft data copies are stored on the researcher’s password-protected laptop. All hard copy surveys are stored in a locked cabinet in the researcher’s office.

## Results

### Demographic data

A total of 100 patients agreed to participate. With an attrition of 38 participants because of missing data, 62 patient participants provided informed consent and completed the surveys. Nine physiotherapist participants were initially recruited and identified for the organisational component. With the attrition of one, eight physiotherapists provided informed consent and completed the organisational component of the survey.

Gender-wise, 48 (77.4%) females and 14 males (22.6%) participated. The majority of participants were aged in the 31-year to 40-year range (32; 51.6%), followed by 11 (17.7%) aged 51 years to 50 years and 10 (16.1%) aged 41 years to 50 years. Fifty-one (82.3%) were employed, 7 (11.3%) were self-employed, 2 (3.2%) were unemployed, 1 was a student and 1 was retired (1.6%). Fifty-nine (95.2%) participants had a tertiary education, and 42 (67.7%) were referred for physiotherapy by doctors. The majority of participants attended the physiotherapy clinic in the Manzini 44 (71.0%) and 18 (29.0%) in the Hhohho region.

Seventy-five per cent of the physiotherapists were female, 5 (62.5%) were between 21 years and 30 years of age and 3 (37.5%) had more than 7 years of working experience.

### Patient-centred barriers

Thirty-four (54.8%) participants had LBP for 0–12 months, 50 (80.6%) had consulted with their physiotherapist at least one to five times, and 32 (51.6%) completed three to five sessions in the consultation that they were currently in (Table 1).

**TABLE 1:** Patient-centred characteristics (*N* = 62).

Patient-centred characteristics	*n*	%
**Duration of low back pain (months)**		
0–12	34	54.8
13–24	8	12.9
25–36	5	8.0
37–48	3	4.8
49–60	1	1.6
> 60	11	17.7
**Number of physiotherapy consultations completed**		
1–5	50	80.6
6–10	9	14.5
> 10	3	4.8
**Missed scheduled physiotherapy appointments**		
No (not missed)	40	64.5
Yes (missed)	22	35.5
**Physiotherapy sessions completed in the current consultation**		
0–2	27	43.5
3–5	32	51.6
> 5	3	4.8
**Low back pain numeric pain rating scale**		
< 5/10	30	48.4
5/10	14	22.6
> 5/10	18	29.0
**Clear understanding of low back pain**		
No (I do not understand)	18	29.0
Yes (I do understand)	44	71.0
**Understanding of how physiotherapy helps low back pain**		
No (I don’t understand)	4	6.5
Yes (I do understand)	58	93.5
**Availability of time in daily schedule to attend physiotherapy**		
No (I do not have time)	14	22.6
Yes (I do have time)	48	77.4

In this study, 15 (24.1%) participants agreed that pain during exercise was a barrier to performing prescribed exercises, 12 (19.4%) agreed that being afraid to do exercises without supervision was a barrier, 11 (17.7%) agreed that they do not have time to do the exercises and 11 (17.7%) forgot to do the exercises and had no reference to follow (Table 2).

**TABLE 2:** Distribution of participants by exercise barriers (*N* = 62).

Exercise barriers	Agree		Disagree	
Barriers to performing prescribed exercises	*n*	%	*n*	%
I do not have time to do the exercises	11	17.7	30	48.4
I am afraid to do the exercises on my own without supervision	12	19.4	40	64.5
The exercises are too many I do not remember some of them	9	14.5	38	61.3
I forget the exercises and I do not have a reference to follow	11	17.7	36	58.1
The exercises are too difficult for me to do at home	3	4.8	23	37.1
My back becomes painful when I do the exercises	15	24.1	29	46.8
I rarely get the opportunity to do the exercises	9	14.5	34	54.8
I do not believe that the exercises will help me	2	3.2	45	72.6
I do not have support from home to help me do the exercises	6	9.7	40	64.5
I do not see any improvement as I do the exercises	5	8.1	44	80.0

Some of the barriers highlighted for not attending set physiotherapy sessions in our study were: 37 (59.7%) agreed that they already felt better, 21 (33.9%) agreed that it was painful, 21 (33.9%) agreed that it was a burden to change their routine and 21 (33.9%) agreed that the cost of continuing with treatment was too much. (Table 3).

**TABLE 3:** Barriers to attending set physiotherapy sessions (*N* = 62).

Barriers to attending set physiotherapy sessions	Agree		Disagree	
	*n*	%	*n*	%
It is a burden to change my routine	21	33.9	27	43.5
I have to keep it a secret	2	3.2	57	91.9
I am disheartened by my condition	16	25.8	37	59.7
There is a huge burden in travelling (distance, cost, accessibility)	7	11.3	45	72.6
I do not see any result	5	8.1	47	75.8
I do not see the importance of doing this	2	3.2	56	90.3
The cost of continuing with treatment is too much	21	33.9	29	47.8
Previous negative experience	1	1.6	58	93.5
It is painful	21	33.9	31	50.0
It is taking too long to resolve	17	27.4	37	59.7
People with this condition are often stigmatised in my society	1	1.6	51	82.3
It does not fit my cultural beliefs	2	3.2	56	90.3
The weather can be unfavourable	8	12.9	47	75.8
I do not believe this can help my condition	8	12.9	54	87.1
I have no support from my family and friends	3	4.8	48	77.4
I already feel better	37	59.7	19	30.6
Forget follow-up appointment	11	17.7	42	67.7

### Provider-centred barriers

Fifty-six (90.3%) patients had prescribed exercises as part of their management routine, 58 (93.5%) reported that clear communication was rendered concerning their condition and treatment procedure, 39 (62.9%) of the patients received a routine follow up, 39 (62.9%) received reminders for upcoming appointments and 51 (82.3%) patients had at most 0–14 days waiting for the next appointment (Table 4).

**TABLE 4:** Provider-centred barriers (*N* = 62).

Provider-centred barriers characteristics	*n*	%
**Exercise prescription by the attending physiotherapist**		
No (they did not prescribe)	6	9.7
Yes (they prescribed)	56	90.3
**Clear communication from the physiotherapist**		
No (it was not clear)	4	6.5
Yes (it was clear)	58	93.5
**Routine follow up by the physiotherapist**		
No (they do not follow up)	23	37.1
Yes (they do follow up)	39	62.9
**Provision of reference material**		
No (they do not provide)	30	48.4
Yes (they provide)	32	51.6
**Reminder of upcoming appointment**		
No (they don’t remind)	23	37.1
Yes (they do remind)	39	62.9
**Waiting period by physiotherapist for follow-up appointment (days)**		
0–14	51	82.3
15–28	1	1.6
29–42	4	6.5
> 43	6	9.7

### Further analysis

‘Too many prescribed exercises’ was significantly negatively associated with adherence to scheduled physiotherapy appointments. Understanding the condition was significantly positively associated with adherence to scheduled physiotherapy appointments (*p* = 0.001). The odds of defaulting scheduled physiotherapy sessions are 6.4 times higher if a patient does not understand the condition or 3 times higher if a patient does not have an exercise prescription. Females more than males, older participants and patients referred to physiotherapy by a doctor are less likely to miss scheduled physiotherapy sessions.

## Discussion

Our pilot study aimed to determine barriers associated with physiotherapy appointment attendance and prescribed home exercise adherence among patients with LBP and the treating physiotherapists in outpatient physiotherapy clinics in Eswatini.

The prevalence of LBP in our study was consistent across cultures but notably higher in females aged 40 years to 69 years, aligning with global trends (Maher et al. 2017; Meucci, Fassa & Xavier Faria 2015; Vos et al. 2015). This demographic is particularly susceptible because of factors such as hormonal changes, higher rates of osteoporosis and occupational roles. Our study highlighted that LBP predominantly affected the employed population, with women being the majority, suggesting that workplace ergonomics and job-related stress are significant contributors (Meucci et al. 2015). The impact of LBP on work and daily activities was profound, underscoring the need for workplace interventions such as ergonomic assessments and modifications (Grabovac & Dorner 2019; Odole & Olugbenga-Alfred 2018).

Interestingly, our findings showed that LBP in Eswatini tends to last up to 12 months for 54.8% of participants, significantly longer than the global average (Popescu & Lee 2020). This prolonged duration could be attributed to limited access to healthcare, cultural beliefs about pain and treatment, and socio-economic factors. Further research is needed to explore these potential causes and develop targeted interventions. Comparatively, a study in Cameroon found a median duration of 33 months for chronic LBP, indicating that prolonged pain may be more common in low- and middle-income countries because of similar barriers to accessing effective treatment (Doualla et al. 2019). Future research should focus on investigating these barriers, implementing workplace ergonomic interventions and understanding the influence of cultural and socio-economic factors on LBP management and outcomes.

Our study revealed that the majority of referrals (67.7%) for physiotherapy treatment came from consulting doctors at the primary healthcare level, with patients being three times more likely to attend sessions if referred by a doctor compared to other sources. This contrasts with findings from a South African study where referrals to physiotherapy were rare and patients were primarily managed with pain medication (Major-Helsloot et al. 2014). Literature indicates that LBP is a major cause of consultations in primary healthcare (Hartvigsen et al. 2018). Similarly, our study showed an increased frequency of physiotherapy consultations, with most participants attending one to five consultations (80.6%) and completing three to five sessions (51.6%). This could be attributed to the recurrent nature of LBP and issues with adherence.

Although non-adherence was reported only by a minority, it is known to cause adverse effects such as recurrent pain. Within physiotherapy, adherence varies, while 64.5% of participants did not miss appointments, 34.5% did, which negatively impacts treatment outcomes (Babatunde et al. 2017) and increases the number of consultations. Barriers to physical activity included a lack of time, pain, fatigue, a lack of visible results and other daily priorities (Mathy et al. 2015). Knowledge of exercises is crucial for long-term adherence to home exercise programmes (Saner et al. 2018). In our study, exercise performance was minimally affected by factors such as lack of time, professional supervision, forgetfulness, difficulty, pain, a lack of improvement and belief that exercise is ineffective. Despite literature highlighting barriers such as fatigue, absence of results, fear of pain, a lack of motivation and increased pain during exercise (Essery et al. 2017; Jack et al. 2010; Palazzo et al. 2016; Van Koppen et al. 2016), the majority of participants in our study disagreed with these barriers.

Although the knowledge of LBP among patients is not well-reported (Barbari et al. 2020), our study found that a majority of participants (71%) understood their condition and an even higher percentage (93.5%) understood how physiotherapy would help them. However, it is noteworthy that 29.0% of patients did not understand their condition, indicating a need for future studies to investigate patients’ knowledge of LBP. Providing clear information about the diagnosis, treatment procedures and follow-up sessions has been shown to positively affect adherence to physiotherapy treatment (Babatunde et al. 2017). In our study, 93.5% of participants received clear communication regarding their condition and treatment strategies, 90.3% were prescribed home exercise programmes and 51.6% received reference material for their exercises. Patients tend to remember their physiotherapists’ advice better when prescribed exercises are simple and few (Bachmann et al. 2018; Supp et al. 2020).

A lack of time among physiotherapists is a significant barrier to managing patients with LBP (Östhols, Boström & Rasmussen-Barr 2019), though current literature does not clearly state if it also affects setting patient treatment sessions. Administrative aspects, such as prior communication to remind patients of appointments, sufficient resources, continuity with the same therapist and reduced waiting times for follow-up appointments, play a crucial role in patient management.

To improve patient adherence in musculoskeletal physiotherapy practice, implementing a therapist-focussed knowledge translation intervention can be highly effective. Babatunde et al. (2017) suggest that brief, interactive educational sessions significantly enhance physiotherapists’ knowledge and confidence in adherence-enhancing activities. In addition, the use of adherence toolkits and behaviour change strategies to address barriers can lead to better patient outcomes. Applying frameworks such as Graham’s knowledge-to-action cycle and continuously evaluating the effectiveness of these interventions are also recommended to refine and improve adherence strategies (Babatunde et al. 2017).

Our pilot study, the first of its kind in Eswatini, highlights the barriers to treatment adherence for LBP in outpatient physiotherapy clinics and identifies provider-centred barriers faced by treating physiotherapists.

### Study limitations and suggestions for future research

The survey was long, potentially leading to incomplete surveys and the attrition rate. However, follow-up communication by the researcher encouraged participation. A cause-and-effect relationship could not be established with the small sample size in our pilot study, but feasibility was established and hence, future research with a larger sample size is advised to establish associations. Future research should also focus on investigating the reasons behind the prolonged duration of LBP in Eswatini, particularly exploring cultural beliefs, socio-economic factors and access to healthcare. In addition, studies should examine the barriers to treatment adherence, including the role of workplace ergonomics and job-related stress. Further research is needed to understand patients’ knowledge of LBP and its impact on treatment outcomes. Implementing and evaluating workplace ergonomic interventions and exploring the administrative and time-related challenges faced by physiotherapists could also provide valuable insights. Finally, developing strategies to enhance patient education and adherence to home exercise programmes should be prioritised to improve overall treatment efficacy.

## Conclusion

Our pilot study identified key barriers to physiotherapy treatment adherence among LBP patients in Eswatini. The prevalence was higher in females aged 40 years to 69 years, predominantly affecting the employed population and impacting daily activities. Low back pain often lasted up to 12 months, longer than global averages, indicating a need for further research.

Doctor referrals significantly increased adherence, although non-adherence, reported by a minority, still negatively impacted outcomes. Barriers included a lack of time, pain, fatigue and a lack of visible results. Despite these, most participants did not miss appointments, showing relatively high adherence.

While 71.0% of participants understood their condition and 93.5% understood physiotherapy benefits, 29.0% lacked this understanding, highlighting the need for better patient education. Clear communication and prescribed home exercise programmes positively influenced adherence.

Physiotherapists’ time constraints and administrative issues were also barriers. Efforts to improve care included appointment reminders, sufficient resources, continuity with one therapist and reduced waiting times. Improving patient education and addressing specific barriers could enhance adherence, potentially reducing the duration and impact of LBP. Implementing a therapist-focussed knowledge translation intervention, as suggested by Babatunde et al. (2017), can significantly improve physiotherapists’ knowledge and confidence in adherence-enhancing activities. Brief, interactive educational sessions, along with the use of adherence toolkits and behaviour change strategies, are effective in addressing barriers to adherence.

In addition, applying frameworks such as Graham’s knowledge-to-action cycle and continuously evaluating the effectiveness of these interventions are essential steps in refining and improving adherence strategies. These approaches collectively contribute to better patient outcomes and more effective physiotherapy practices. One significant finding is the crucial role of prescribed home exercise programmes in improving patient outcomes. Despite challenges such as lack of time, pain and other priorities, adherence is improved by providing clear communication and simple, easy-to-remember exercises. Reference material provision and follow-up sessions further support patient commitment to exercises, leading to functional recovery and better pain management. By implementing targeted interventions and continuously evaluating effectiveness, adherence to home exercise programmes can be achieved with improved outcomes for patients with LBP. Our study is the first in Eswatini to explore these barriers in outpatient physiotherapy clinics.
